# Supplementary material for: Automatic quantification of tumor-stroma ratio as a prognostic marker for pancreatic cancer
Source: PLoS One. 2024 May 21;19(5):e0301969. doi: 10.1371/journal.pone.0301969 (PMC11108171; doi:10.1371/journal.pone.0301969)
Supplement: Attachment — Submitted filename: Response.docx [file pone.0301969.s001.docx]

Dear Mia Vanessa Recto,

I am answering to your email to address the following question:

1) Thank you very much for your submission to PLOS ONE. Before we proceed, we kindly ask that you address the discrepancy in the ethics documentation. The individuals, de Groot and Geachte mevrouw Nagtegaal, named as investigators on the ethics documents are not the authors on the present submission. As we would generally expect the investigator named on the ethical approval to be an author on the submission, can you please provide an explanation for this situation?

The reason why the investigator named on the ethical approval are not listed as authors is that the data was collected in a study by de Groot and Prof. Nagtegaal, while this study is a secondary use of the data that is allowed by the ethical committee approval.


Should you have further questions, please do not hesitate to contact me.

Best regards,

Pierpaolo Vendittelli
